# Muscle strength mediates the relationship between mitochondrial energetics and walking performance

**DOI:** 10.1111/acel.12568

**Published:** 2017-02-09

**Authors:** Ariel C. Zane, David A. Reiter, Michelle Shardell, Donnie Cameron, Eleanor M. Simonsick, Kenneth W. Fishbein, Stephanie A. Studenski, Richard G. Spencer, Luigi Ferrucci

**Affiliations:** ^1^ Translational Gerontology BranchNational Institutes of HealthBaltimoreMDUSA; ^2^ Laboratory of Clinical InvestigationIntramural Research Program, National Institute on AgingNational Institutes of HealthBaltimoreMDUSA

**Keywords:** 31P MRS, bioenergetics, *in vivo*, muscle strength, skeletal muscle, walking speed

## Abstract

Skeletal muscle mitochondrial oxidative capacity declines with age and negatively affects walking performance, but the mechanism for this association is not fully clear. We tested the hypothesis that impaired oxidative capacity affects muscle performance and, through this mechanism, has a negative effect on walking speed. Muscle mitochondrial oxidative capacity was measured by *in vivo* phosphorus magnetic resonance spectroscopy as the postexercise phosphocreatine resynthesis rate, k_PC__r_, in 326 participants (154 men), aged 24–97 years (mean 71), in the Baltimore Longitudinal Study of Aging. Muscle strength and quality were determined by knee extension isokinetic strength, and the ratio of knee extension strength to thigh muscle cross‐sectional area derived from computed topography, respectively. Four walking tasks were evaluated: a usual pace over 6 m and for 150 s, and a rapid pace over 6 m and 400 m. In multivariate linear regression analyses, k_PC__r_ was associated with muscle strength (β = 0.140, *P *=* *0.007) and muscle quality (β = 0.127, *P *=* *0.022), independent of age, sex, height, and weight; muscle strength was also a significant independent correlate of walking speed (*P *<* *0.02 for all tasks) and in a formal mediation analysis significantly attenuated the association between k_PC__r_ and three of four walking tasks (18–29% reduction in β for k_PC__r_). This is the first demonstration in human adults that mitochondrial function affects muscle strength and that inefficiency in muscle bioenergetics partially accounts for differences in mobility through this mechanism.

## Introduction

Walking independently is essential for performing daily living activities and maintaining independence throughout the human lifespan (Halter *et al*., 2009). Preferred walking speed often slows with increasing age in older adults (Cunningham *et al*., 1982), and this metric of lower extremity performance is a strong predictor of disability, functional dependence, and mortality in the adult and older population (Guralnik *et al*., 1995; Studenski *et al*., 2011; Schrack *et al*., 2012). As walking is a complex task requiring energy, balance, movement control, and coordination of the musculoskeletal, nervous, respiratory, and cardiovascular systems, it is difficult to evaluate the extent to which loss of mobility with aging can be attributed to any single factor (Ferrucci *et al*., 2016).

In a previous study conducted on participants drawn from the Baltimore Longitudinal Study of Aging (BLSA), we demonstrated that skeletal muscle mitochondrial oxidative capacity (as measured by *in vivo* phosphorus magnetic resonance spectroscopy, ^31^P MRS, in the quadriceps) is a strong independent predictor of walking speed, and this association between oxidative capacity and walking speeds is especially strong for more challenging gait tasks (Choi *et al*., 2016). These findings suggested that diminished bioenergetic reserve (Schrack *et al*., 2013) and/or impaired bioenergetic synthetic capacity is in part responsible for slower walking speeds in older persons. This hypothesis is consistent with the progressive decline in muscle mass and strength (with the decline in strength outpacing that of muscle mass) (Goodpaster *et al*., 2006; Newman *et al*., 2006) often observed in older persons and attributed in part to higher prevalence of chronic diseases (McDermott *et al*., 2004) and lower physical activity in older compared with younger persons (Cesari *et al*., 2009).

Studies conducted in animal models and in humans have shown that skeletal muscle mitochondrial oxidative capacity declines with age (Conley *et al*., 2000; Short *et al*., 2005; Fleischman *et al*., 2010; Peterson *et al*., 2012), likely due to a decline in both total mitochondrial mass and decreased intrinsic mitochondrial functional capacity (Conley *et al*., 2000). This steady decline of mitochondrial function with aging is believed to contribute to the progressive deterioration of muscle strength and quality (Metter *et al*., 1999), as diminished energy production may constrain muscle performance, and dysfunctional mitochondria create oxidative stress that can damage proteins and mitochondrial DNA (Peterson *et al*., 2012). Thus, it is reasonable to hypothesize that the association between decreased skeletal muscle bioenergetics synthetic capacity and slower walking speed shown in our previous study is mediated by the deleterious effect of impaired oxidative capacity on muscle performance; that is, that lower mitochondrial function relates to lower muscle strength, which in turn relates to slower walking speed. We hypothesize that this pathway explains, in part, the link between muscle mitochondrial function and walking speed.

Muscle mitochondrial function can be studied by *in vivo* ^31^P MRS, a noninvasive technique used to assess the rate of maximum *in vivo* oxidative capacity of skeletal muscle by quantitatively measuring phosphorus‐containing metabolites—phosphocreatine (PCr), inorganic phosphate (P_i_), and adenosine triphosphate (ATP) (Chance *et al*., 1981; Edwards *et al*., 2012). This method is reproducible (McCully *et al*., 2009), can be focused to a specific, single muscle (McCully *et al*., 1993), is not affected by postural or balance impairments, and has been validated against *in vitro* techniques (McCully *et al*., 1993; Conley *et al*., 2000). Muscular oxidative capacity is indexed by the postexercise PCr resynthesis rate constant, or k_PCr_, which is determined by monitoring the mono‐exponential recovery of PCr (Meyer, 1988) in the rest period following an exercise‐induced depletion. This rate constant, k_PCr_, has been established as a measure of mitochondrial ATP production (McCully *et al*., 1993, 2009; Conley *et al*., 2000; Edwards *et al*., 2012; Coen *et al*., 2013).

This cross‐sectional study uses data from the BLSA to test the hypothesis that this relationship is mediated, at least in part, by the age‐related decline in muscle strength. Specifically, we examined whether k_PCr_ is an independent predictor of muscle strength, and of walking speed in four different walking tasks (Coen *et al*., 2013; Choi *et al*., 2016). Then, we performed a formal mediation analysis to evaluate whether the association between mitochondrial function and walking speed is mediated by impaired muscle strength.

## Results

**Figure 1:**
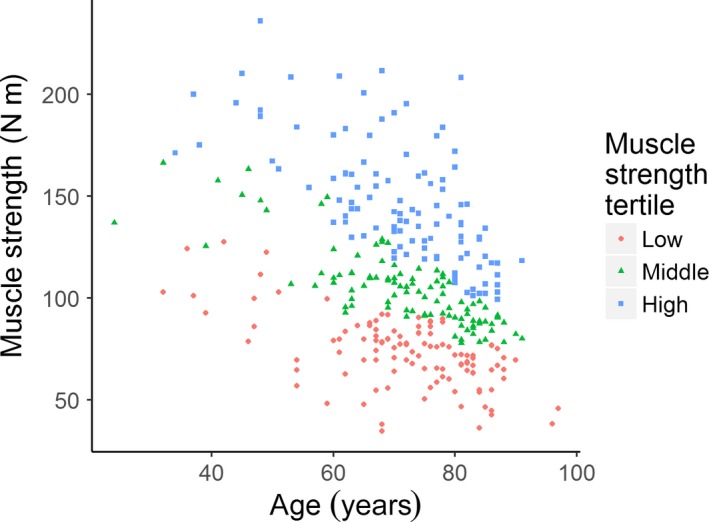
Age versus muscle strength stratified by age‐specific muscle strength tertiles, for 326 participants in the Baltimore Longitudinal Study of Aging.

The cross‐sectional study sample included 326 participants, 47% men, with a mean age of 71.4 years (range 24–97 years). Figure 1 shows, as expected, that there is an overall decline of muscle strength with age. To compare individuals with differences in maximum quadriceps muscle strength independent of age, we stratified the study population by age decades, dividing participants within each decade according to strength tertiles before cumulating them across age decades. The three groups so identified are shown as indicated in different colors (Fig. 1); their descriptive summary characteristics are compared in Table 1. By design, age did not differ across the three strength groups (Table 1). Maximum quadriceps muscle strength was lower in women than in men (i.e., women made up 86% of the low muscle strength tertile), and height, weight, and thigh muscle cross‐sectional area were higher across muscle strength tertiles (*P *<* *0.001 for all; Table 1). Further, the median k_PCr_ was higher with increasing muscle strength (*P *=* *0.036), and % PCr depletion was also different across the groups (Table 1), with greater depletion corresponding to greater strength. Four walking tasks were tested: UGS‐6 m (usual gait speed, for a distance of 6 m), UGS‐150 m (usual gait speed, 150 m), RGS‐6m (rapid gait speed, 6 m), and RGS‐400m (rapid gait speed, 400 m). The average walking speeds were faster with increasing muscle strength (*P *<* *0.01 for all).

**Table 1:** Distribution of selected characteristics of the 326 participants from the Baltimore Longitudinal Study of Aging, according to age‐adjusted muscle strength tertile [given as mean (SD)]

Characteristic		Muscle strength tertiles (N·m, 30° per s)			*P*‐value
		Low	Middle	High	
	*n*	111	106	109	
Age (years)	326	71.63 (12.98)	71.74 (12.59)	70.75 (12.26)	0.820
Sex (female)	326	96 (86%)	57 (54%)	19 (17%)	<0.001^e^, ^b^
Height (cm)	326	161.17 (8.54)	168.22 (7.92)	173.30 (7.97)	<0.001^e^, ^c^
Weight (kg)	326	68.20 (15.29)	75.48 (14.10)	82.96 (12.43)	<0.001^e^, ^c^
kPCr (per s)^a^	326	0.019 (0.006)	0.020 (0.007)	0.021 (0.006)	0.036^e^, ^d^
% PCr depletion	326	34.69 (10.05)	36.66 (11.41)	41.58 (11.60)	<0.001^e^, ^c^
Thigh muscle cross‐sectional area (cm^2^)	326	85.77 (23.48)	102.27 (27.46)	125.91 (26.40)	<0.001^e^, ^c^
Usual gait speed, UGS‐6m (m s^−1^)	316	1.16 (0.19)	1.22 (0.21)	1.24 (0.21)	0.009^e^, ^c^
Usual gait speed, UGS‐150m (m s^−1^)	316	1.20 (0.14)	1.22 (0.16)	1.26 (0.15)	0.009^e^, ^c^
Rapid gait speed, UGS‐6m (m s^−1^)	316	1.75 (0.28)	1.85 (0.34)	1.91 (0.37)	<0.001^e^, ^c^
Rapid gait speed, UGS‐400m (m s^−1^)	316	1.45 (0.20)	1.49 (0.24)	1.58 (0.25)	<0.001^e^, ^c^

a Expressed as: median (IQR).

b Fisher's exact test *P*‐value.

c Adjusted for age and sex.

d Jonckheere–Terpstra test *P*‐value.

e Statistically significant

After adjustment for age, sex, height, weight, and % PCr depletion, k_PCr_ was significantly and positively associated with maximum quadriceps muscle strength considered as a continuous variable (*P *=* *0.007) and muscle quality (calculated as the ratio of left quadriceps peak torque at 30° per s to left thigh muscle cross‐sectional area, N·m cm^−2^, *P *=* *0.022), but not thigh muscle cross‐sectional area (*P *=* *0.141, Table 2). These analyses were based on the 326 participants who had complete information for spectroscopy and muscle characteristics. As subsequent analyses were based on the subgroup of individuals who also had data on walking speed, we repeated the analyses reported in Table 2, limited to the 316 participants with complete data, and obtained results that were substantially similar to the original (Table S2, Supporting information).

**Table 2:** Linear regression models for k_PCr_ rates predicting left thigh muscle cross‐sectional area, muscle strength, and muscle quality, adjusted for age, sex, height, and weight, and corrected for % PCr depletion. All coefficients are standardized

*N* = 326, male = 154						
Parameters	Muscle area (cm^2^)		Muscle strength (N·m)		Muscle quality (N·m cm^−2^)	
Adj. *R* ^2^	0.765		0.531		0.183	
	β (95% CI)	*P*‐value	β (95% CI)	*P*‐value	β (95% CI)	*P*‐value
Age	−0.367 (−0.427, −0.306)	<0.001^a^	−0.313 (−0.398, −0.228)	<0.001^a^	−0.270 (−0.139, 0.085)	0.633
Sex	0.543 (0.467, 0.619)	<0.001^a^	0.326 (0.219, 0.433)	<0.001^a^	−0.188 (−0.330, −0.046)	0.010^a^
Height (cm)	−0.098 (−0.190, −0.006)	0.037^a^	0.212 (0.082, 0.341)	0.002^a^	0.414 (0.243, 0.585)	<0.001^a^
Weight (kg)	0.430 (0.357, 0.504)	<0.001^a^	0.066 (−0.037, 0.170)	0.210	−0.374 (−0.511, −0.238)	<0.001^a^
kPCr (per s)	0.044 (−0.014, 0.102)	0.141	0.114 (0.032, 0.197)	0.007^a^	0.127 (0.019, 0.236)	0.022^a^
% PCr dep.	−0.005 (−0.061, 0.051)	0.857	0.217 (0.138, 0.296)	<0.001^a^	0.323 (0.219, 0.427)	<0.001^a^

a Statistically significant

For all four walking tasks (UGS‐6 m, UGS‐150s, RGS‐6m, and RGS‐400m), k_PCr_ was a significant independent predictor of walking speed (*P* < 0.05 for all) when adjusting for age, sex, height, weight, and % PCr depletion (Table 3). In agreement with previous findings (Choi *et al*., 2016), we found that this association was strongest for the most challenging task, RGS‐400m (β = 0.208, *P *<* *0.001). While Choi *et al*. did not find a significant association between k_PCr_ and UGS‐6 m (the least challenging gait task), in this larger sample, this association became statistically significant, and remained significant after adjusting for confounders. In separate linear regression models adjusted for age, sex, height, and weight, maximum quadriceps muscle strength alone was found to be a significant, independent correlate of walking speed in all four walking tasks, with the association again being the strongest for the most challenging task, RGS‐400m (β = 0.350, *P* < 0.001). Of note, when we introduced into the statistical models an index variable distinguishing the data analyzed in the previous paper (period = 0), and those added to the analytical sample later on (period = 1), and we tested for a k_PCr_*period interaction, this term was never statistically significant, suggesting that our data did not substantially change after expanding the sample size (*P* > 0.10, see Statistical analyses section). In all analyses, relationships between k_PCr_ and muscle strength were stronger than analogous relationships between k_PCr_ and muscle quality. Therefore, subsequent mediation analyses and walking speed models only included muscle strength as a predictor, and not muscle quality.

**Table 3:** Linear regression models (*N* = 316 for all) for each walking speed (WS) task, showing the association between muscle strength (MS) and walking speed (Model 1), and the effect of k_PCr_ on walking speed, without (Model 2) and with (Model 3) accounting for muscle strength. All models are adjusted for age, sex, height, weight, and % PCr depletion, and all coefficients are standardized

Parameters	Model 1: WS = MS		Model 2: WS = kPCr		Model 3: WS = kPCr + MS	
	β (95% CI)	*P*‐value	β (95% CI)	*P*‐value	β (95% CI)	*P*‐value
Walking Speed = UGS‐6 m (m s^−1^)						
Age	−0.278 (−0.396, −0.161)	<0.001^a^	−0.305 (−0.418, −0.192)	<0.001^a^	−0.251 (−0.371, −0.130)	<0.001^a^
kPCr (per s)	–	–	0.122 (0.012, 0.232)	0.030^a^	0.102 (−0.009, 0.213)	0.072
% PCr depletion	–	–	0.031 (−0.075, 0.136)	0.568	−0.008 (−0.118, 0.101)	0.879
Muscle strength, MS (N·m)	0.180 (0.042, 0.319)	0.011^a^	–	–	0.175 (0.031, 0.320)	0.018^a^
Walking Speed = UGS‐150s (m s^−1^)						
Age	−0.148 (−0.270, −0.026)	0.018^a^	−0.198 (−0.316, −0.080)	0.001^a^	−0.118 (−0.243, 0.006)	0.064
kPCr (per s)	–	–	0.140 (0.025, 0.255)	0.018^a^	0.110 (−0.004, 0.225)	0.060
% PCr depletion	–	–	0.045 (−0.065, 0.155)	0.424	−0.012 (−0.126, 0.101)	0.830
Muscle strength, MS (N·m)	0.261 (0.118, 0.405)	<0.001^a^	–	–	0.257 (−0.107, 0.407)	0.001^a^
Walking Speed = RGS‐6m (m s^−1^)						
Age	−0.379 (−0.485, −0.273)	<0.001^a^	−0.440 (−0.543, −0.337)	<0.001^a^	−0.361 (−0.470, −0.252)	<0.001^a^
kPCr (per s)	–	–	0.104 (0.003, 0.205)	0.044^a^	0.074 (−0.025, 0.174)	0.145
% PCr depletion	–	–	0.088 (−0.009, 0.184)	0.075	0.031 (−0.068, 0.129)	0.544
Muscle strength, MS (N·m)	0.273 (0.149, 0.398)	<0.001^a^	–	–	0.256 (0.126, 0.387)	<0.001^a^
Walking Speed = RGS‐400m (m s^−1^)						
Age	−0.452 (−0.549, −0.355)	<0.001^a^	−0.510 (−0.605, −0.415)	<0.001^a^	−0.408 (−0.506, −0.310)	<0.001^a^
kPCr (per s)	–	–	0.208 (0.115,0.301)	<0.001^a^	0.170 (0.080, 0.259)	<0.001^a^
% PCr depletion	–	–	0.094 (0.005, 0.183)	0.039^a^	0.020 (−0.069, 0.109)	0.657
Muscle strength, MS (N·m)	0.350 (0.236, 0.465)	<0.001^a^	–	–	0.330 (0.212, 0.447)	<0.001^a^

a Statistically significant

Further analyses were aimed at testing the hypothesis that the relationship of mitochondrial function with muscle strength was mediated by a pro‐inflammatory state or a status of impaired glucose metabolism. In preliminary analyses, we found nonsignificant age‐, sex‐, height‐, weight‐ and PCr depletion‐adjusted partial correlations of k_PCr_ with fasting glucose (*r* = −0.029, *P* = 0.619), fasting insulin (*r* = −0.084, *P* = 0.151), 2‐h glucose after an oral glucose tolerance test (*r* = −0.048, *P* = 0.407), and IL‐6 (*r* = −0.085, *P* = 0.152). In addition, multivariate regression models predicting maximum quadriceps muscle strength (adjusting for the same covariates) did not substantially alter the size and significance of the effect of k_PCr_ on maximum quadriceps muscle strength (data not shown).

In linear regression models that included both k_PCr_ and muscle strength, for all four walking tasks, the relationship between k_PCr_ and walking speed was attenuated by the inclusion of muscle strength (with a reduction in the magnitude of the β coefficient ranging from 16 to 29%) and only remained significant for RGS‐400m (β = 0.170, *P *<* *0.001). In contrast, muscle strength remained statistically significant for all four walking speed outcomes, with estimated coefficients decreasing only by 2–6% when accounting for k_PCr_. This mediation effect was confirmed by significant (*P *<* *0.040) Sobel test statistics for three of the four walking speed tasks: UGS‐150 m indirect effect of 0.894 (95% CI = 0.18, 1.872), RGS‐6m indirect effect 1.993 (95% CI = 0.447–4.040), RGS‐400m indirect effect 1.816 (95% CI = 0.461–3.442), with muscle strength as a mediator explaining 18–29% of the influence of k_PCr_ on these three walking tasks. The Sobel test statistic for the least challenging task, UGS‐6 m, was not significant (*P *=* *0.075, indirect effect = 0.835, CI 0.072–1.950), although muscle strength was calculated to account for 17% of the effect of k_PCr_ on walking speed even in this case.

## Discussion

This study shows an association between an *in vivo* ^31^P MRS‐measured index of skeletal muscle mitochondrial oxidative capacity and four measures of walking speed in the largest sample to date (*n* = 316). Adjusting for age, sex, height, weight, and % PCr depletion, higher oxidative capacity was positively associated with faster walking speeds for all four walking tasks, reinforcing the proposition that a progressive deterioration of muscle bioenergetics with aging exerts influence on gait speeds (Coen *et al*., 2013) and that muscle mitochondrial oxidative capacity may be especially rate‐limiting for tasks requiring more exertion (Choi *et al*., 2016).

**Figure 2:**
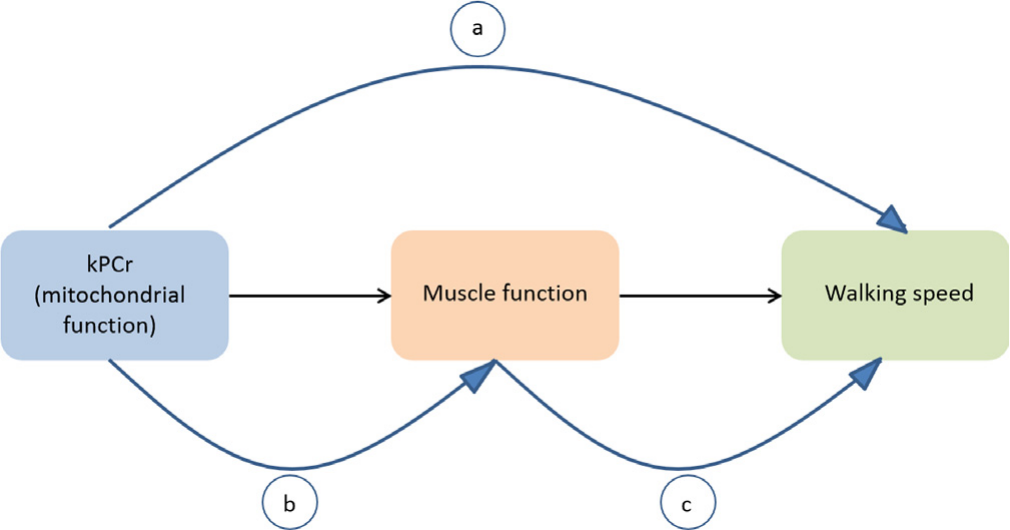
Diagram illustrating: a) the demonstrated effect of k_PC__r_ on walking speeds (Choi *et al*., 2016), b) the effect of k_PC__r_ on muscle function, and c) the effect of k_PC__r_ on walking speeds, mediated by muscle function.

To further explore the mechanism by which mitochondrial oxidative capacity affects walking speeds, in the present study, we tested the hypothesis that the relationship between k_PCr_ and walking speed outcomes is mediated by, at least in part, muscle strength (Fig. 2). We showed that independent of potential confounders, k_PCr_ is significantly correlated with muscle strength and quality. Several mechanisms may account for this association. First, decreasing mitochondrial oxidative capacity with age results from the combination of decreasing mitochondrial volume and increasing mitochondrial dysfunction (Peterson *et al*., 2012). Mitochondrial dysfunction creates oxidative stress by generating reactive oxygen species (ROS) that can damage mitochondrial DNA (Peterson *et al*., 2012), impair calcium regulation, and affect myofilament structure and function (Powers *et al*., 2011). These would all act to decrease skeletal muscle force production. An additional possibility is that diminished ATP synthetic capacity could affect the efficiency of the skeletal muscle cross‐bridge cycle, which is coupled to the myosin ATPase‐controlled hydrolysis of ATP and is responsible for skeletal muscle force production (Holmes & Geeves, 2000).

In a previous study, Fleischman and collaborators similarly tested the association between ^31^P muscle bioenergetics and maximal voluntary contraction of the knee extensor muscle (Fleischman *et al*., 2010). They found that this association was no longer significant when adjusted for age and covariates. However, the participants evaluated by Fleischman and collaborators were children (8–17 years) and middle‐aged adults (18–55 years) (Fleischman *et al*., 2010); the relationship between mitochondrial function and muscle strength may be different in prepubescent children, and in this population, the degree of age‐related mitochondrial impairment may also be small and unlikely to critically affect muscle contraction. This is consistent with the decline in aerobic fitness being relatively modest in young and middle age, likely due to a relative stability of mitochondrial volume and function (Short *et al*., 2005; Peterson *et al*., 2012).

In contrast, we studied a much older population that included persons with relatively poor muscle strength and slower walking speeds or impaired gait capability. In this population, even a single muscle contraction may be an energetic challenge that is sensitive to even minor reductions in muscle oxidative capacity. In this sense, our findings are consistent with those of Bourdel‐Marchasson and collaborators, who show that muscle mitochondrial function is related to global physical functional status, particularly in hospitalized and frail older subjects (Bourdel‐Marchasson *et al*., 2007). Our findings are also consistent with recent reports in the literature that associate cumulative mitochondrial DNA polymorphisms and circulating mitochondrial DNA copy number with frailty (Moore *et al*., 2010). Of interest, the strong association between k_PCr_ and muscle strength, but not muscle mass (Table 2), suggests that impaired mitochondrial function and efficiency may account for some of the discrepancy between age‐related decline in muscle strength (dynapenia) and the age‐related decline in muscle mass (sarcopenia) (Manini & Clark, 2012).

When muscle strength was included along with k_PCr_ in the models predicting walking speed, the relationship between walking speed and k_PCr_ was attenuated, with a decrease of up to 29% in the k_PCr_ coefficients, and with loss of statistical significance in all but the most challenging gait task, RGS‐400m (Table 3). This suggests that impaired overall muscle function accounts for a significant portion of the association between mitochondrial oxidative capacity and walking speed. However, in the most strenuous walking task, this mediation effect was substantially attenuated. This indicates that at this higher level of physiologic stress, mitochondrial function plays a central role not just through its effect on muscle strength, but also more directly, through the role of maintaining an adequate flux of energy during the entire task (Schrack *et al*., 2013). Indeed, this finding is consistent with recent hypotheses that impaired mitochondrial function constitutes part of the etiology of elevated fatigability in older individuals (Santanasto *et al*., 2014), which may contribute to slower gait and loss of mobility (Vestergaard *et al*., 2009).

Our findings are based on the largest population ever studied using *in vivo* ^31^P MRS as a measure of mitochondrial oxidative capacity (k_PCr_), In addition, we have incorporated multiple state‐of‐the‐art measures of health and functional status from a well‐characterized population. However, our study also has limitations. We were only able to study participants who were eligible for and able to complete a thigh CT scan, the ^31^P MRS exercise protocol, isokinetic dynamometry, and four walking speed tasks, all of which may exclude some of the frailest individuals and those with impaired mobility. In addition, the BLSA is mostly comprised of a relatively healthy and well‐educated cohort of volunteers, which may not be representative of the aging population at large.

The magnitude of the association between mitochondrial energetics and mobility performance explained by muscle strength or quality (i.e., the extent of mediation) is relatively small. This is consistent with the notion that age‐associated mobility decline is multifactorial. Nevertheless, our findings remain important both for the fundamental understanding of the physiology of muscle aging and because of the potential development of exercise and drug therapies to improve mitochondrial function.

In addition, although the development and progression of chronic disease presumably plays a role in the overall loss of mobility and physical function with increasing age, we did not include an adjustment for chronic diseases in our analyses. Diseases such as diabetes also have specific associations with mitochondrial dysfunction (Sivitz & Yorek, 2010), and the influence of chronic disease on the causal pathway to mobility loss remains to be elucidated. In contrast, habitual physical exercise exerts a positive influence on mitochondrial volume (Brierley *et al*., 1996). It would be of interest to examine the relationship between habitual physical activity and measured muscle mitochondrial oxidative capacity in a future study, to further separate out components and influences of fitness and exercise on both overall muscle function and intrinsic mitochondrial capacity. Finally, this study was cross‐sectional, and therefore, we could not fully exclude the possibility of reverse causality—namely, the possibility that reduced mobility causes muscle impairment and, in turn, mitochondrial dysfunction. Longitudinal data on these variables are currently collected in the BLSA.

Overall, our results show that skeletal muscle bioenergetics influence functional mobility as defined by walking speed and that this influence is in part mediated by muscle strength and function. We found that this mediation begins to break down for the most demanding walking tasks, with bioenergetics playing a more significant direct role at that level of activity. Thus, interventions aimed at improving muscle mitochondrial function, as well as maintaining muscle strength and quality, may reduce, postpone, or prevent mobility loss. Physical exercise is the only intervention thus far that has been shown to significantly improve muscle strength in older individuals (Latham *et al*., 2004). In addition, there is literature support for the positive role of resistance exercise and elevated levels of aerobic physical activity on mitochondrial content and function in the elderly (McCully *et al*., 1993; Brierley *et al*., 1996; Jubrias *et al*., 2001), presenting a coherent picture with our current results. Together, these studies provide strong support for exploration of such interventions as an important prescriptive step in retaining functional mobility and independence with aging.

## Experimental procedures

### Participants

The design, study population, and measurement protocols of the BLSA have been documented previously (Shock *et al*., 1984). Established in 1958, the BLSA is a continuous enrollment cohort study of community‐dwelling adults conducted by the National Institute on Aging Intramural Research Program. Participants with no major chronic conditions or functional impairments are enrolled in the study and follow‐up visits occur at intervals of 1‐4 years, becoming more frequent for older participants. Baseline data on the ^31^P postexercise recovery rate (k_PCr_) were collected from 438 BLSA participants from April 2013 to September 2015. Of these, 326 participants (154 men), aged 24–97 years (mean 71.4 ± 12.6 years), had complete datasets including a physical examination, a health history questionnaire, isometric knee extensor strength testing, and thigh CT scans; 316 participants also had completed data on walking speed tasks for the same visit. As expected, participants who had information on muscle but not on walking performance (*n* = 10) had significantly lower muscle mass and strength (Table S1, Supporting information). Trained and certified technicians administered all assessments, following standardized protocols. Height and weight were measured according to standard protocols, and the average of three measures was used in the analysis. The National Institute of Environmental Health Sciences Institutional Review Board approved the study protocol, and all participants gave written informed consent.

### Gait speed metrics

The four gait tasks performed were identical to those outlined previously (Choi *et al*., 2016). The usual and rapid short‐course gait speed measurements were performed over a 6‐m course; participants were asked to first walk at their ‘normal walking pace’ (UGS‐6 m), then ‘as fast as possible’ (RGS‐6m); the fastest of two trials was used in the analyses. The long‐distance usual and rapid gait speed tasks are components of the Long Distance Corridor Walk, which uses a 20‐m course in an uncarpeted corridor (Simonsick *et al*., 2001). The first walk (UGS‐150s) was performed at the participant's ‘usual, comfortable pace’ for 150 s (2.5 min). The second walk (RGS‐400m) follows immediately, with the participant walking 400 m ‘as quickly as possible’.

### Muscle strength, mass, and quality

Maximum quadriceps muscle strength was defined as the highest of three consecutive values of torque (N·m) measured by left leg concentric knee extensor contraction at an angular velocity of 30° per s using an isokinetic dynamometer (Biodex Multi‐Joint System‐PRO with Advantage Software V.4X, Biodex Medical Systems, Inc., Shirley, NY, USA; Hartmann *et al*., 2009). This torque value is equal to the force generated during the knee extension, multiplied by the length from the knee to the point where the dynamometer is applied on the tibia. Thigh muscle cross‐sectional area was also measured for the left leg, from 10‐mm slice thickness computed topography (CT) images acquired at midfemur (Somatom Sensation 10; Siemens, Malvern, PA, USA) and quantified by customized software with manual checking for quality control (geanie software, version 2.1; BonAnalyse, Jyvaskyla, Finland). Density thresholding was used to separate fat from muscle tissue (at a threshold of 35 mg mm^−1^) and to separate muscle from bone tissue (at a threshold of 180 mg mm^−1^). In addition, any intramuscular fat that was macroscopically detectable was also excluded from the calculation of muscle area. Muscle quality was defined as the ratio of maximum left quadriceps peak torque (N·m) to thigh muscle cross‐sectional area (cm^2^) (Moore *et al*., 2014).

### Oral glucose tolerance test

For the oral glucose tolerance test (OGTT), participants fasted for 10 h overnight, and fasting plasma was collected at baseline (0 min). Participants then drank a 75‐g glucose solution and plasma samples were drawn every 20 min for 120 min. Glucose levels were quantified with a glucose oxidase analyzer (YSI Incorporated, Yellow Springs, OH, USA), and plasma insulin was assessed by enzyme‐linked immunosorbent assay (ELISA) (Mercodia Inc., Winston‐Salem, NC, USA). Complete OGTT data at baseline and at 2 h were collected during the same visit as the ^31^P MRS, gait speed, and maximum quadriceps muscle strength testing for 300 participants. Participants on insulin are excluded from the test.

### IL‐6 quantification

Blood serum samples were assayed to assess cytokine levels. IL‐6 concentrations were quantified using ELISA kits (R&D System, Minneapolis, MN, USA), with intra‐ and interassay variations of 1.6–4.2% and 0.3–6.4%, respectively. Complete IL‐6 data were collected for 293 participants with same‐visit data for all other measurements.

### ^31^P MRS

The ^31^P muscle bioenergetics measurement protocol was identical to that outlined previously (Choi *et al*., 2016). *In vivo* spectra of ^31^P‐containing metabolites were acquired using a 3T Philips Achieva MR scanner (Philips, Best, The Netherlands) and a 10‐cm ^31^P‐tuned surface coil (PulseTeq, Surrey, UK) fastened over the left thigh vastus lateralis muscle. Participants performed a rapid ballistic knee extension exercise while lying supine in the bore of the magnet; this maneuver was practiced before entering the magnet (Coen *et al*., 2013; Choi *et al*., 2016). A series of pulse‐acquire ^31^P spectra were obtained before, during, and after the knee extension exercise. The pulse sequence consisted of adiabatic RF excitation pulses with a 90‐degree flip angle. A total of 75 dynamic acquisitions were performed, with TR = 1.5 s, and the raw data consisted of spectra obtained at 1.5‐s temporal resolution. Signals were averaged over four successive acquisitions for SNR enhancement, so that the data consisted effectively of spectra obtained with a temporal resolution of 6 s (i.e., a 6‐s interval between consecutive time points and a total scan duration of 7.5 min). The length of exercise was monitored to achieve between a 33 and 66% reduction in PCr peak height and never exceeded 42 s, with a postexercise recovery period of 5.8–6.3 min; spectra were processed using jmrui (version 5.0) and quantified using a nonlinear least squares algorithm (AMARES; Vanhamme, Vanhamme *et al*., 1999; Naressi *et al*., 2001).

### Skeletal muscle oxidative ATP resynthesis rate determined by ^31^P MRS

The recovery rate for phosphocreatine was calculated by fitting the postexercise time‐dependent change in PCr to a mono‐exponential function of the form:

(1) $$\text{PCr}\left(t\right)\;=\;{\text{PCr}}_{0}\;+\;\Delta \text{PCr}\left(1-\text{exp}\left(-t/\tau_{\text{PCr}}\right)\right)$$

where PCr_0_ is the end‐of‐exercise PCr signal area (i.e., the PCr signal area at the beginning of the recovery period), ΔPCr is the decrease in signal area from its pre‐exercise baseline value of PCr_baseline_ to PCr_0_ resulting from in‐magnet exercise, and τ_PCr_ is the PCr exponential recovery time constant. The inverse of τ_PCr_, k_PCr_ (= 1/τ_PCr_), is thus the recovery rate constant. This is taken as an index of *in vivo* muscle oxidative phosphorylation capacity, as there are minimal other energy demands during this resting period; postexercise PCr resynthesis is considered primarily a function of maximum mitochondrial ATP production with no or minimal contribution of anaerobic metabolism (Arnold *et al*., 1984; McCully *et al*., 1993, 2009; Conley *et al*., 2000; Edwards *et al*., 2012). The % PCr depletion (Walsh *et al*., 2001; Smith *et al*., 2004) was determined by calculation of the decrease in the PCr peak area from pre‐exercise PCr_baseline_ to PCr_0_; this use of area is distinct from the use of PCr peak height in real‐time monitoring of bioenergetics status during exercise. The intramuscular pH was also monitored (calculated according to the chemical shift of inorganic phosphate, P_i_, relative to PCr) to avoid acidosis and ensure that intramuscular pH did not drop below 6.80 (Arnold *et al*., 1984).

### Statistical analyses

The distributions of population characteristics were compared across age‐adjusted tertiles of muscle strength using analysis of variance, the Fisher exact test, and the Jonckheere–Terpstra test for trend.

The cross‐sectional relationships between age, sex, height (cm), weight (kg), k_PCr_ (per s), % PCr depletion, and outcomes of muscle area, muscle strength, and muscle quality were evaluated in linear regression models; all linear regression coefficients were standardized so as to be comparable. Possible nonlinearity in the relationships between mitochondrial function and muscle area, muscle strength, and muscle quality was investigated by adding a quadratic term for k_PCr_ to the final models; this quadratic term was not found to be statistically significant for any of these outcomes. Similarly, an interaction term between sex and k_PCr_, when added to the final models, was also not statistically significant (*P* > 0.10). The % PCr depletion term was included as an adjustment to control for exercise intensity among participants (Walsh *et al*., 2001; Smith *et al*., 2004). In addition, to address the combined use of new observations (*n* = 243) with previously published older ones (*n* = 83, from Choi *et al*., 2016), corresponding interaction terms were tested in all of the final models and were found not to be statistically significant (*P* > 0.05). By including an indicator for ‘new data’, and testing its interaction with k_PCr_ in predicting the outcome of interest, separate models for each temporal set were essentially fit at once. The lack of statistically significant interactions indicates the consistency of the two datasets, as expected, as they were acquired with identical experimental protocols and analyses.

To test whether the relationship of k_PCr_ with maximum quadriceps muscle strength was mediated by a pro‐inflammatory state or impaired glucose metabolism, we tested the partial correlations between k_PCr_ and baseline glucose, baseline insulin, 2‐h glucose after OGTT, and IL‐6 levels, individually, after adjusting for age, sex, height, weight, and PCr depletion. None of these were found to be statistically significant, and including these same variables in multivariate linear regression models predicting maximum muscle quadriceps strength did not markedly attenuate the magnitude or significance of the effect of k_PCr_.

To evaluate the relationship between muscle strength, k_PCr_, and the four different walking speed outcomes, we fit a set of linear regression models for each walking task, adjusting for sex, height, weight, and % PCr depletion in every model. For each walking task (Table 3), Model 1 demonstrates the effect of muscle strength on walking speed. Model 2 estimates the coefficient linking k_PCr_ and walking speed, and Model 3 adds muscle strength to Model 2 to evaluate changes in the size of the coefficient linking k_PCr_ to walking speed after adjusting for muscle strength. The Sobel test was performed to evaluate whether muscle strength mediates the effect of k_PCr_ on walking speed for all four walking tasks. We also computed the proportion of the association between k_PCr_ and walking speed that is explained by muscle strength. In addition, k_PCr_^2^ and sex–k_PCr_ interaction terms were tested and as they did not contribute significantly to the models’ fit, these terms were removed from the models. All analyses were performed using r version 3.3.1 (R Foundation for Statistical Computing, Vienna, Austria), and *P *<* *0.05 was considered statistically significant for all analyses.

## Funding

This research was supported entirely by the Intramural Research Program of the National Institutes of Health, National Institute on Aging.

## Author contributions

ACZ, DAR, DC, KWF, SAS, RGS, and LF were involved in the study conception, design, and/or experimental protocol; ACZ, DAR, MS, DC, EMS, and LF performed the collection of and/or analysis of data; ACZ and LF wrote the manuscript. All authors approved the final version of the manuscript.

## Conflict of interest

The authors have no conflict of interest to declare.

## Supporting information


**Table S1** Selected characteristics given as mean (SD), comparing the excluded participants who were missing walking task data (*n* = 10), with those who had completed walking task data.
**Table S2** Linear regression models for k_PCr_ rates predicting left thigh muscle cross‐sectional area, muscle strength, and muscle quality, adjusted for age, sex, height, and weight, and corrected for % PCr depletion (as in Table 2) for the reduced sample size, *n* = 316.
