# Supplementary material for: Muscle strength mediates the relationship between mitochondrial energetics and walking performance
Source: Aging Cell. 2017 Feb 9;16(3):461–8. doi: 10.1111/acel.12568 (PMC5418194; doi:10.1111/acel.12568)
Supplement: Supplementary file 1 — Table S1 Selected characteristics given as mean (SD), comparing the excluded participants who were missing walking task data (n = 10), with those who had completed walking task data. Table S2 Linear regression models for kPCr rates predicting left thigh muscle cross‐sectional area, muscle strength, and muscle quality, adjusted for age, sex, height, and weight, and corrected for % PCr depletion (as in Table 2) for the reduced sample size, n = 316. [file ACEL-16-461-s001.docx]

**Supplementary Material**

Table S1. Selected characteristics given as mean (sd), comparing the excluded participants who were missing walking task data (n = 10), with those who had completed walking task data. The full study population (n = 326) characteristics are also included for reference.

|  | Full set |  | Excluded vs. included | |  |
| --- | --- | --- | --- | --- | --- |
| Characteristic | Baseline cohort  n = 326 |  | Did not complete walking tasks n = 10 | Completed walking tasks n = 316 | *p*-value^*^ |
| Age (years) | 71.37 (12.68) |  | 77.40 (13.11) | 71.18 (12.54) | 0.124 |
| Sex (female) | 172 (53%) |  | 8 (80%) | 164 (52%) | 0.109^‡^ |
| Height (cm) | 167.52 (9.55) |  | 161.38 (9.65) | 167.65 (9.50) | 0.002^†^ |
| Weight (kg) | 75.50 (15.22) |  | 73.93 (19.97) | 75.55 (15.08) | 0.697^†^ |
| kPCr (s^-1^) | 0.021 (0.005) |  | 0.018 (0.006) | 0.021 (0.005) | 0.106^†^ |
| % PCr depletion | 37.64 (11.38) |  | 34.13 (7.74) | 37.75 (11.47) | 0.321^†^ |
| Thigh muscle cross sectional area (cm^2^) | 104.56 (30.61) |  | 81.37 (17.78) | 105.29 (30.66) | < 0.001^†^ |
| Muscle strength (N·m) | 108.09 (38.24) |  | 84.86 (29.88) | 108.83 (38.28) | 0.009^†^ |

^*^ all between the excluded and included groups
^†^adjusted for age and sex
^‡^ Fisher Exact test *p*-value

Table S2. Linear regression models for k_PCr_ rates predicting left thigh muscle cross-sectional area, muscle strength, and muscle quality, adjusted for age, sex, height and weight, and corrected for % PCr depletion (as in Table 2) for the reduced sample size, n = 316. All coefficients are standardized. The results do not suggest a significant departure from the models using n = 326.

| n = 316, male = 152 | | | | | | |
| --- | --- | --- | --- | --- | --- | --- |
| Parameters | Muscle Area (cm^2^) | | Muscle Strength (Nm) | | Muscle Quality (Nm/cm^2^) | |
| Adj. R^2^ | 0.765 | | 0.531 | | 0.183 | |
|  | β (95% CI) | *p*-value | β (95% CI) | *p*-value | β (95% CI) | *p*-value |
| Age | -0.360 (-0.420, -0.301) | < 0.001* | -0.309 (-0.396, -0.223) | < 0.001* | -0.024  (-0.137, 0.089) | 0.673 |
| Sex | 0.528 (0.452, 0.604) | < 0.001* | 0.317 (0.208, 0.427) | < 0.001* | -0.184 (-0.327, -0.040) | 0.013* |
| Height (cm) | -0.103 (-0.193, -0.012) | 0.028* | 0.210  (0.079, 0.341) | 0.002* | 0.412 (0.240, 0.583) | < 0.001* |
| Weight (kg) | 0.455 (0.381, 0.529) | < 0.001* | 0.081 (-0.026, 0.188) | 0.138 | -0.376 (-0.516, -0.236) | < 0.001* |
| kPCr (s^-1^) | 0.050  (-0.008, 0.108) | 0.094 | 0.116 (0.031, 0.200) | 0.007* | 0.120  (0.009, 0.229) | 0.035* |
| % PCr dep. | -0.001 (-0.057, 0.055) | 0.969 | 0.224 (0.143, 0.304) | < 0.001* | 0.325  (0.219, 0.430) | < 0.001* |
